# Biofortified Maize Can Improve Quality Protein Intakes among Young Children in Southern Ethiopia

**DOI:** 10.3390/nu11010192

**Published:** 2019-01-18

**Authors:** Nilupa S. Gunaratna, Debebe Moges, Hugo De Groote

**Affiliations:** 1 Department of Nutrition Science and Public Health Graduate Program, Purdue University, West Lafayette, IN 47907, USA; 2 Applied Human Nutrition, School of Nutrition, Food Science and Technology, Hawassa University, P.O. Box 5, Hawassa, Ethiopia; debebe2m@gmail.com; 3 International Maize and Wheat Improvement Center (CIMMYT), P.O. Box 1041-00621, Nairobi, Kenya; h.degroote@cgiar.org

**Keywords:** biofortification, protein quality, dietary assessment, seasonality, Ethiopia

## Abstract

Quality protein maize (QPM) varieties are biofortified, or nutritionally improved, to have higher lysine and tryptophan levels to increase quality protein intakes particularly among young children. This study assesses adequacy of children’s protein intakes in Ethiopia, where QPM is being promoted, accounting for protein quality and seasonal dietary changes, and estimates potential increases in intakes if QPM replaced conventional maize in diets. Diets of randomly sampled children aged 12–36 months in rural southern Ethiopia (*n* = 218) were assessed after harvest during relative food security and 3–4 months later during relative food insecurity using 24-h weighed food records. Diets were analyzed for protein adequacy, accounting for protein quality using the protein digestibility corrected amino acid score (PDCAAS) method, and potential improvements from QPM substitution were estimated. Stunting was prevalent (38%) at the first assessment. Across seasons, 95–96% of children consumed maize, which provided 59–61% of energy and 51–55% of total protein in 24 h. Dietary intakes decreased in the food insecure season, though children were older. Among children no longer breastfeeding, QPM was estimated to reduce inadequacy of utilizable protein intakes from 17% to 13% in the food secure season and from 34% to 19% in the food insecure season. However, breastfed children had only 4–6% inadequate intakes of utilizable protein, limiting QPM’s potential impact. Due to small farm sizes, maize stores from home production lasted a median of three months. Young Ethiopian children are at risk of inadequate quality protein intakes, particularly after breastfeeding has ceased and during food insecurity. QPM could reduce this risk; however, reliance on access through home production may result in only short-term benefits given the limited quantities of maize produced and stored.

## 1. Introduction

Genetic biofortification, in which the nutritional contribution of food crops is increased through plant breeding, is an agricultural strategy to reduce nutrient deficiencies, particularly among rural populations in developing countries [1]. This strategy requires that a crop biofortified with a specific nutrient is targeted to a population that is both deficient in that nutrient and likely to consume the biofortified crop in sufficient quantities to reduce the deficiency. However, for many nutrients, such as protein, it is not clear in which populations deficiencies exist, especially when the target is young children for whom dietary data are often limited [2]. Furthermore, even if a nutritional deficiency exists in a population, the biofortified crop may not necessarily alleviate that deficiency as its impact depends on individuals’ sustained access to the biofortified crop and consumption as part of a regular diet.

The first commercial biofortified crop was quality protein maize (QPM), a set of maize varieties developed through conventional plant breeding to have grain with improved protein quality [3]. This was achieved through higher grain levels of the essential amino acids lysine and tryptophan, obtained after crossbreeding with “high-lysine maize”, which had a natural genetic mutation that altered the amino acid profile of its grain (Table S1) [4]. Subsequent plant breeding efforts have increased the lysine and tryptophan content of QPM varieties, while also improving its agronomic and consumer characteristics [3,5].

Randomized, controlled studies in which QPM was provided to rural households in diverse settings with specific encouragement to use the varieties to feed young children at risk of undernutrition found that QPM could improve child nutritional status [6]. Specifically, in Ethiopia, two randomized, controlled studies found that home cultivation and use of QPM for child feeding could reduce or prevent growth faltering in community settings and may in some cases support catch-up growth in weight [7]. QPM varieties are available to farmers at similar prices as conventional maize and do not require special growing conditions; QPM plant breeding efforts have also addressed farmers’ concerns about the pre- and post-harvest performance of the earlier high-lysine maize [3]. Consumer acceptance studies in Latin America and East Africa have found acceptance and sometimes preference for QPM over conventional maize varieties for the preparation of common traditional foods [8,9,10], including preference by rural Ethiopian mothers for QPM in the preparation of complementary foods [11]. As a result, QPM varieties are being promoted in many developing countries where maize is a staple food [10]. In Ethiopia, where child undernutrition is widespread with large health and economic consequences [12,13], the government has set specific targets to increase cultivation of QPM in the near term.

Overall, dietary quality is poor in Ethiopia, and young children typically do not consume foods that provide high quality protein [14]. A recent study found that stunting among Ethiopian children was highly prevalent and that linear growth failure in these children was likely associated with low intakes of quality protein and energy [15]. In Southern Ethiopia, a cross-sectional study found all participating pregnant women in their third trimester had inadequate protein intakes, consuming a median of 15.5 g of utilizable protein per day, consistent with low biochemical measures [16]. In the same region, when assumptions were made regarding breast milk intake, children aged 6–23 months had low though adequate total protein intakes on average, with median intakes of 3.3 g/day among 6–8 month olds, 3.6 g/day among 9–11 month olds, and 7.3 g/day among 12–23 month olds [17]. However, that analysis did not account for decreased utilization when protein quality is poor [18], and utilizable protein intakes may therefore also be inadequate among young children in this region.

While maize is widely cultivated in Southern Ethiopia and QPM varieties are actively promoted, it remains to be shown that on-farm production and home consumption of these varieties can improve the adequacy of protein intakes among young children in this region. Limited data exist on the diets of young children, the adequacy of protein in those diets, and the contribution of maize, and specifically of home-grown maize, to those diets. Most QPM target areas further experience highly seasonal food insecurity, which can affect children’s diets and dietary quality as well as access to home-grown maize over time. Therefore, in this paper, the adequacy of protein intakes is assessed among young children in Southern Ethiopia, where QPM is currently being promoted, taking into account protein quality and seasonal changes in diet. A simulation exercise is conducted to estimate the potential increase in quality protein intakes if conventional maize in children’s diets were replaced by QPM.

## 2. Materials and Methods

### 2.1. Conceptual Framework

For a biofortified crop to have a positive impact on nutrition, the prevalence of inadequate intakes of the target nutrient must be sufficiently high to be of public health importance in the target population. Both intakes and requirements vary among individuals in a population, and the prevalence of inadequacy is the proportion of individuals in a population whose intakes are below their requirements for a given nutrient [19]. For young children, protein requirements depend on body weight and age, with average requirements of 0.95 g/kg body weight/day at 12 months, 0.85 g/kg body weight/day at 18 months, 0.79 g/kg body weight/day at 24 months, and 0.73 g/kg body weight/day at 36 months [18]. Furthermore, these requirements increase when children experience energy deficits or acute or chronic infections, which are common risks in poor and food insecure populations [18].

Meanwhile, an individual’s intake of a specific nutrient comes from the crop that may be biofortified as well as all other components of the total diet. Consequently, biofortification efforts target populations consuming diets with low diversity and low consumption of other foods that are good sources of the nutrient of interest. In the case of protein, such good sources would include animal source foods, legumes, and nuts. For children who are breastfeeding, breastmilk can be an important source of quality protein and other nutrients. The average quantity and nutrient content of consumed breastmilk varies by child age and location, with distinctions made between developed and developing countries [20]. In many developing country populations, particularly those that rely significantly on subsistence agriculture, diets and therefore nutrient intakes vary seasonally, along with seasonal changes in the availability and access to specific foods. The quality of protein in a complete diet depends on the digestibility of the protein sources and their essential amino acid composition relative to a reference pattern that depends on age [18]. Correction of total protein intake to account for these two factors yields an estimate of utilizable protein, i.e., the net amount of protein utilized by the body.

Biofortified crops are largely expected to have nutritional impact through home production and consumption [21]. This approach requires that sufficient amounts of the biofortified crop will be cultivated, reserved for home consumption, stored, and consumed over sufficient duration to realize nutritional impact. Consequently, rural semi-subsistence households are often targeted. The crop that is biofortified must sufficiently contribute to the total diet so that substitution of a non-biofortified variety with a biofortified variety will markedly increase intake of the target nutrient and meaningfully reduce an existing nutrient deficiency. Biofortification efforts therefore focus on staple crops. The increase in target nutrient intake depends on the quantity of the biofortified crop that is consumed, the increase in the nutritional contribution of the crop through biofortification, and the degree of maintenance of the nutritional improvement during cultivation, storage, processing, and food preparation. It is further assumed that with adoption of a biofortified crop, agricultural and dietary practices will remain largely unchanged, i.e., a biofortified variety will take the place of a non-biofortified variety already used for home production and consumption. If there is already sufficient production and consumption of a target crop, such a substitution could result in nutritional impact. However, if the crop does not already play a major role in home production or consumption, additional behavior change will be required to realize impact.

### 2.2. Study Overview

In this study, diets of children aged 12–36 months were assessed at two points in time in rural southern Ethiopia. Biofortification primarily relies on home production and consumption; therefore, maize was both the main crop and the main dietary staple in the study area. As diets were hypothesized to vary seasonally, the first assessment was conducted a few months after the maize harvest at a time of relative food security. The second assessment was conducted three to four months later at a time of increasing food insecurity. The diets were analyzed for protein adequacy, and the potential improvement in protein adequacy was estimated if conventional maize in the diet were hypothetically substituted with QPM.

### 2.3. Study Area and Population

This study was conducted in four communities (known as peasant associations or *kebeles*) in a rural district, Habela Tula, located approximately 15 km south of the city of Hawassa in the Sidama Zone of the Southern Nations, Nationalities, and Peoples’ Region (SNNPR), Ethiopia. This district was a target area for the project, “Quality Protein Maize Development for The Horn and East Africa”, which was led by the International Maize and Wheat Improvement Center (CIMMYT) and aimed to improve food security, nutrition, health, and incomes of resource-poor farming households by developing and facilitating the adoption of QPM varieties [5]. Agriculture was the main source of livelihood in this district, and maize was the major food crop and dietary staple, though QPM was not yet cultivated in this area at the time of the study.

While QPM can potentially benefit a person of any age, these varieties are primarily targeted towards young children with the aim of improving growth and nutritional status. Therefore, the target age range of 12–36 months included the second year of life when children are particularly vulnerable to growth faltering and the third year when growth faltering often continues and children are increasingly dependent on solid foods [22]. However, the first year of life, when children are highly or exclusively dependent on breastfeeding, was excluded.

### 2.4. Study Design

Immunization records maintained at the local community-level health posts in each of the four study communities were used to create a sampling frame of children aged 12–36 months, from which 218 children were selected through simple random sampling. To avoid within-household correlations, only one child per household participated in the study. If two children within the target age range were randomly selected from the same household, one child was selected at random to participate. Children were excluded from study participation if they were visibly ill during recruitment.

The sample size of 218 was determined to allow at most a 7% margin of error for any estimated prevalence or proportion at a given time point, assuming 95% confidence and a 10% non-response rate [23]. Households were visited twice for data collection. The first visit was conducted in January–February 2010, a relatively food secure period following the main maize harvest, which typically falls in September–October. The same households were visited again in May–June 2010, a less food secure period during which cultivation of the next season’s crop had begun but green maize was not yet available for consumption. At each time point, weekdays and weekend days were proportionally represented to capture variation in diets over the seven-day week.

### 2.5. Data Collection

Trained female research assistants, who were from the study communities and fluent in the local language, conducted all informed consent and data collection at study participants’ homes. At each visit, the research assistant remained in the household from 6:00 a.m. to 8:00 p.m. to assess the selected child’s diet using a one-day weighed food record [24]. As it does not rely on participants’ recall, weighed food records provide a high degree of accuracy in quantitatively estimating food and nutrient intakes. All foods and beverages consumed by the child were weighed using a digital scale accurate to the nearest 1 g (model CS2000, OHAUS Corporation, Parsippany, NJ, USA). Detailed weighed recipe data were collected for all composite dishes consumed by the child and used to calculate the weight of actual ingredients consumed [25]. When actual recipe data were not available, average recipe data were compiled. The number of times the child was observed to be breastfeeding during the observation day was also recorded, and the number of times the mother recalled breastfeeding from 8:00 p.m. the previous night to 6:00 a.m. of the observation day was similarly recorded. At the first household visit, the mother or female caregiver of the selected child was additionally administered a questionnaire on household demographic and socioeconomic characteristics and breastfeeding and complementary feeding practices for the selected child. Also, at the first household visit, child weight and height (or recumbent length for children under 24 months) were measured in duplicate using calibrated equipment and standard techniques [24].

### 2.6. Data Analysis

All analyses were conducted using SAS version 9.4 (SAS Institute Inc., Cary, NC, USA). Anthropometric data on children were used to calculate standardized scores for height/length-for-age (HAZ), weight-for-height (WHZ), and weight-for-age (WAZ), based on the 2006 World Health Organization (WHO) Child Growth Standards [26]. Stunting, acute malnutrition, and underweight were defined as HAZ, WHZ, and WAZ respectively more than two standard deviations below the corresponding WHO reference median.

At each assessment, consumption of the following food groups was determined for each child: grains, roots, and tubers; legumes and nuts; dairy products; animal flesh foods; eggs; vitamin A-rich fruits and vegetables; and other fruits and vegetables. Dietary diversity was defined as the number of food groups consumed in a 24-h period [27]. A food composition database was compiled using data from Ethiopia [28,29], supplemented with data from the USDA National Nutrient Database for Standard Reference, Release 28 [30]. Protein digestibility of each item was obtained from published sources [31,32,33]. Energy, protein, and essential amino acid intakes were calculated from the weighed food records, and utilizable protein intakes were then calculated using the protein digestibility corrected amino acid score (PDCAAS) method [18].

As breastfeeding was assessed only during the first household visit, children who were breastfeeding during that visit were conservatively assumed to be breastfeeding during the second household visit (i.e., there was no cessation of breastfeeding between the two household visits). As the duration of individual feedings was not assessed, all children who breastfed at least once in the 24-h period were assumed to have consumed an average amount of breastmilk for their given age. Specifically, estimated nutrient intake from breastmilk was based on existing literature on women and children from developing countries (5.8 g/day for 12–23-month-olds) [20], and breastfeeding children aged 24 or more months were assumed to have half the nutrient intake from breastmilk as children aged 12–23 months. Protein from breastmilk was considered completely utilizable. The prevalence of inadequacy for protein was then estimated as the proportion of intakes below their corresponding age-appropriate estimated average requirement (EAR) for protein [19], as provided by WHO/FAO/UNU and given above [18].

To simulate the potential impact of QPM in this population, utilizable protein intake and prevalence of inadequacy were recalculated assuming all maize in children’s diets was completely substituted with QPM. The improvement in protein quality in QPM is due to increased concentrations of lysine and tryptophan in QPM grain. These concentrations are approximately doubled in QPM compared with conventional maize varieties, and the specific increase varies among QPM varieties [3]. However, this increase may be partially lost during cultivation when QPM plants are cross-pollinated by non-QPM plants, resulting in non-QPM grain, or may be diluted during harvest, storage, processing, or food preparation when QPM grain is mixed with non-QPM grain, from which it is visually indistinguishable. Therefore, simulation of the potential impact of QPM on utilizable protein intake and adequacy conservatively assumed an 80% increase in lysine and tryptophan concentrations compared with conventional maize. This simulation also assumed that no other aspects of the diet would change with the substitution of conventional maize with QPM.

### 2.7. Ethics

Prior to the start of data collection, each mother or caregiver who participated in the study provided written informed consent for themselves and their children, or provided a fingerprint in cases of illiteracy, as witnessed by a local community health worker. This study was approved by the Ethical Review Committee at Hawassa University. Analysis of de-identified data was approved by the Institutional Review Board (IRB) at the Harvard T.H. Chan School of Public Health.

## 3. Results

### 3.1. Participant Characteristics

In the study area, households were large, with nearly six members on average (Table 1). Both household heads, who were mostly male, and caregivers to children, who were mostly female, had limited education, and caregivers had roughly half the years of formal schooling as household heads. Poverty was high, with the majority of homes lacking improved roofs or walls, and one in three households lacked a toilet. Households had limited land (0.4 ha on average), which was primarily used to grow maize (0.3 ha on average). The majority of households also grew small amounts of cash crops such as coffee or khat (*Catha edulis*), though cultivation of more nutritious food crops such as vegetables was less common (38%).

**Figure 1:**
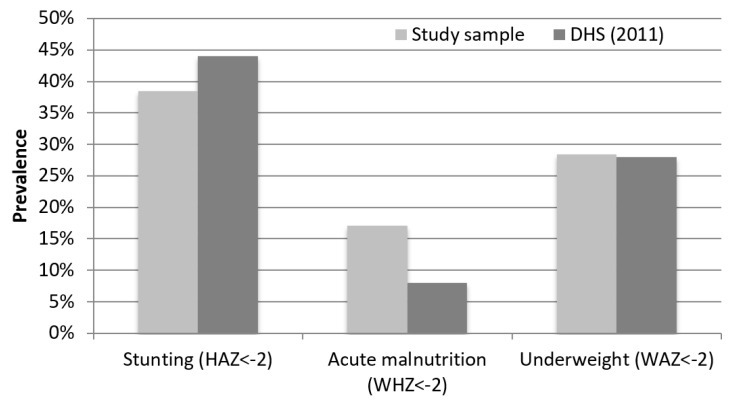
Child nutritional status at the first assessment (post-harvest, *n* = 218), compared with children under five years in the Southern Nations, Nationalities, and Peoples’ Region (SNNPR) reported in the 2011 Ethiopia Demographic and Health Survey (DHS) [14]. HAZ: height-for-age z-score; WHZ: weight-for-height z-score; WAZ: weight-for-age z-score.

At the first assessment, one in three households was already relying on food not produced on-farm. Evaluated children were 25 months old on average. Most children (78%) were still breastfeeding, including 99% of children under 24 months, 78% of children aged 24–29 months, and 52% of children aged 30–36 months. Child undernutrition was highly prevalent with 38% of sampled children stunted (Figure 1), compared with 44% of children under five years stunted in SNNPR in the Ethiopia Demographic and Health Survey (DHS) conducted the following year [14]. Acute malnutrition was twice as high in the study sample (17%) than in the 2011 Ethiopia DHS (8%).

### 3.2. Characteristics of Children’s Diets

Children’s dietary diversity was low (2.3–2.4 out of 7 food groups consumed in a 24-h period) and consistent across the two assessments (Table 2). In a given day, almost all children (95–96%) consumed maize, with the majority (63–65%) also consuming other starchy staples. The majority (61–68%) also consumed dairy products, but consumption of vegetables, rich in vitamin A (40–48%) or otherwise (21–25%), was lower. Very few children consumed legumes or nuts (2–6%), and no children in the study sample consumed eggs or animal flesh foods (meat, fish, poultry, or organs) at either assessment. At each assessment, maize was a regular part of children’s diets and contributed more than half of their energy (59–61%) and total protein (51–55%) intakes (Table 3). Even though children were 3–4 months older at the second assessment, their energy and total protein intakes from complementary feeding both decreased by 23%, likely reflecting a reduction in food security.

### 3.3. Adequacy of Protein Intake and Potential Impact of QPM

Only 72–73% of total protein from complementary foods was utilizable (Table 4), illustrating the importance of correcting for protein quality when analyzing diets that have low digestibility or few sources of quality protein. At a population level, accounting for protein from breastmilk among children who were still breastfeeding increased average utilizable protein intakes by 26% at the first assessment and 33% at the second assessment. At the first assessment, which was conducted in a relatively food secure period, this reduced the estimated prevalence of inadequate protein intake from 28% to 6%. At the second assessment, which was conducted in a more food insecure period, the estimated prevalence of inadequate protein intake was higher: 38% when complementary foods alone were considered, and 12% when estimated breastmilk intake at population level was also considered.

Meanwhile, substituting conventional maize with QPM among children who consumed maize increased average utilizable protein intakes by 8–12%, depending on assessment and whether breastmilk intake was considered. When breastmilk intake was not considered, hypothetical substitution with QPM reduced the estimated prevalence of inadequate protein intake from 28% to 17% at the first assessment and from 38% to 25% at the second assessment. When estimated breastmilk intake at population level was included, substitution of conventional maize with QPM reduced prevalence of inadequate protein intake from 6% to 5% at the first assessment. The change, from 12% to 6%, was greater at the second assessment, when there was greater food insecurity. 

**Figure 2:**
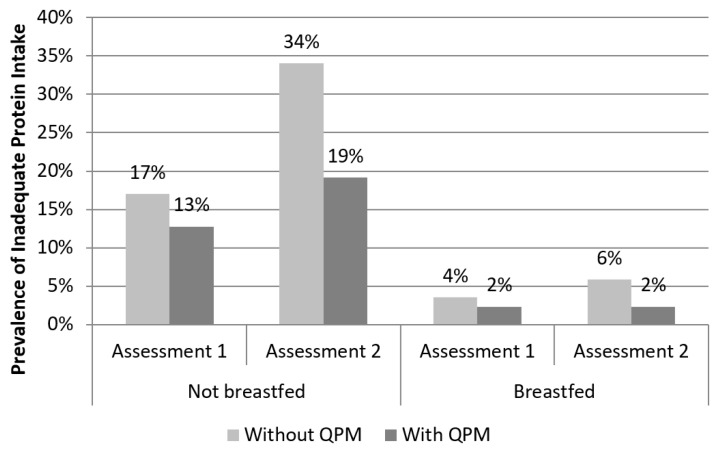
Prevalence of inadequate protein intake with and without quality protein maize (QPM) substitution, disaggregated by child breastfeeding status.

More specifically, when children were disaggregated by breastfeeding status, one in six (17%) older children who had ceased breastfeeding were estimated to have inadequate protein intakes at the first assessment, and this figured doubled (34%) as food insecurity increased at the second assessment (Figure 2). With the assumptions made about the quantity and quality of breastmilk, younger children who were still breastfeeding had only 4% prevalence of inadequacy, which increased to only 6% in the more food insecure season. Substitution of conventional maize with QPM was estimated to reduce the risk of inadequacy by 25% (from 17% to 13%) among older, non-breastfed children at the first assessment and by 44% (from 34% to 19%) at the second assessment. Meanwhile, among younger, breastfed children, QPM was estimated to have limited impact, reducing the already low prevalence of inadequacy from 4% to 2% at the first assessment and from 6% to 2% at the second assessment.

**Figure 3:**
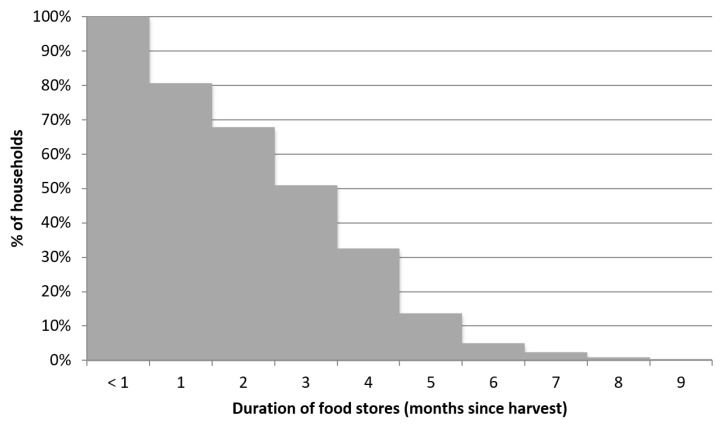
Distribution of the duration of household food stores.

Finally, likely due to limited land available for crop cultivation, households were able to store harvested food for at most nine months (Figure 3). The median household depleted its food stores three months after harvest. One in five households (19%) stored harvested food for less than one month after harvest.

## 4. Discussion

To our knowledge, this is the first study to model the potential impact of a biofortified crop using quantitative dietary data from a target population across seasons. Rural young Ethiopian children were at risk of inadequate protein intakes, particularly after the quality of that protein was taken into account. Given the role of maize as a staple food, substitution of conventional maize with QPM in children’s diets could significantly reduce this risk. While the target of many nutritional and nutrition-sensitive interventions is children in the first two years of life, older children who had ceased breastfeeding were significantly more likely to have inadequate protein intakes and greater potential benefit from the introduction of QPM. As is common in many rural areas reliant on subsistence agriculture, food security and diets were seasonal, and children’s food—and specifically protein intakes—were significantly reduced as food insecurity increased, resulting in greater risk of inadequate protein intakes and greater potential benefit of QPM. However, impact of a biofortified crop requires concurrent nutritional need and access to the crop for a meaningful duration, and in this region, access to home-grown staples was also seasonal. Reliance on access to biofortified foods through home production alone may result in limited impact because of the limited quantity of crops produced and stored.

Households in the study area were rural and poor, with high levels of child undernutrition typical of the region. Young children had monotonous diets with few sources of quality protein: legumes were rarely consumed and, aside from dairy, animal foods were not consumed at all. Maize was a staple in both agricultural production and children’s diets, motivating the promotion of QPM in this area. Dietary assessment using weighed food records from a representative sample of young children, including children beyond the first two years of life, over two time points was a strength of this study, providing a quantitative look at protein intakes in a target population across seasons. While dietary diversity can be a simple and quick indicator of micronutrient adequacy in a diet [34], in this study, dietary diversity remained unchanged while energy and protein intakes decreased significantly across seasons. This indicated the value of quantitative dietary assessment and suggested that food insecurity, at least in the short term, manifested itself in reduced intake rather than changes in the types of food consumed. There were no major events affecting food security in the study area over the study duration; the change in dietary intakes over time therefore reflected the typical seasonality of diets in this region. Furthermore, the two assessments were conducted approximately three months apart in a region with a single crop season per calendar year. It is therefore possible that children’s intakes were higher soon after the crop harvest and even lower later in the season, prior to the next harvest.

Protein intakes among young children in the study area were low and lowered further after adjustment for protein quality. Adjustment is necessary for these and similar diets with low intake of animal source foods; otherwise, protein intake is overestimated and protein inadequacy underestimated. The PDCAAS method used in this study to assess protein quality has the known limitation of using fecal amino acid digestibilities of foods rather than ileal digestibilities, which would more accurately reflect the quantity of amino acids absorbed [35]. However, this methodology cannot be improved in practice until data on ileal amino acid digestibilities are available for common foods in the Ethiopian diet.

Young children in this study were at risk of inadequate protein intakes particularly if they had ceased breastfeeding or during periods of relative food insecurity. While it has been widely held that children globally receive sufficient dietary protein [36], recent research suggests that shortfalls may exist and may be associated with linear growth faltering [37]. Current estimates of protein requirements do not address children’s protein needs for optimal linear growth; increased protein and amino acid requirements due to frequent infections, growth faltering, or energy deficit; or the roles of protein and amino acids in growth regulation and immune function [38,39,40]. Adjusting for increased protein requirements due to recurring infections and energy deficits significantly increased estimates of the prevalence of inadequate protein supply in developing countries [41]. In this target population, with its prevalent infections, energy deficits, and need for catch-up growth, adjusting for increased protein requirements will leader to higher estimated prevalence of inadequate protein intakes and potentially greater estimated benefit from QPM.

Ex ante impact assessments of biofortified crops typically use a disability-adjusted life year (DALY) framework and national-level data [42], making several assumptions about the diets of target populations and without considering seasonality of either diets or potential impact. The use of quantitative dietary data from a specific target population was a strength of this study. Substitution of conventional maize with QPM was estimated to improve dietary intake of quality protein in young Ethiopian children, and the impact was greater among non-breastfed children, particularly in seasons of relative food insecurity. This suggests that while children are considered more nutritionally vulnerable in the first 1000 days of life [43], in this population, their nutritional need may be greater when they are older and no longer consume breastmilk. Likewise, QPM and potentially other biofortified crops may be more beneficial for older children. Meanwhile, breastfed children may have higher utilizable protein intakes and may therefore not benefit significantly from QPM. However, this depends on several assumptions about the quantity and nutritional quality of consumed breastmilk, and these assumptions may not hold in populations such as in this study, which have prevalent maternal undernutrition and prolonged breastfeeding [44].

The estimated effect of QPM introduction also depends on several assumptions. In this study, the nutritional contents of foods in available food composition tables were conservatively used; however, several factors in rural Ethiopia and similar environments may reduce the protein and essential amino acid content of common foods, and this would likely reduce overall utilizable protein intake and increase the potential impact of QPM. For example, when grown on low nitrogen soils, many crops including maize, the staple food in this region, will have lower protein content [45], which is unlikely to be reflected in food composition tables. Injera, a common food throughout Ethiopia that is typically made with teff (*Eragrostis tef*), is often made with a mixture of teff and maize, the latter of which has lower protein quality, as maize grain is cheaper and therefore more accessible to the poor. Finally, unpublished data from one of the authors (DM) found that milk samples taken from households in the study area were diluted with water by 10–25%.

The potential benefit of QPM also depends on the increase in lysine and tryptophan content achieved through the introduction of the quality protein trait. This increase depends on multiple genetic systems and varies among different varieties of the same biofortified crop, and there are active efforts to boost the increase in lysine and tryptophan content through plant breeding [3,10]. However, this increase can later be lost or diluted on farms, depending on adopters’ pre- and post-harvest management practices, food processing, and preparation, and infant and young child feeding practices. These multiple barriers, which may ultimately reduce target children’s consumption of QPM with the full quality protein trait, may require additional interventions to address household behaviors and practices [46].

In this study, the estimated effect of QPM was also calculated based on a single weighed food record from each participant at each time point. This is a limitation as calculated protein intakes therefore included both variation in usual intakes among children in the population and day-to-day variation in intakes for a given child. Usual intakes alone would give more accurate estimates of the prevalence of inadequacy, but, to separate out within-child variation in intakes, more than one observation per participant at each time point would be required for at least a subset of participants [19]. However, given the monotonous diets observed among children, it may be reasonable to assume that within-child variation in protein intakes is also relatively low in this population and therefore has limited impact on the estimated prevalence of inadequate protein intakes with or without QPM.

For QPM or any biofortified crop to have nutritional impact, target individuals must have sustained access to that crop. Biofortified crops are largely intended to be accessed through home production, storage, and consumption, and in many rural areas of the developing world, access to foods produced on-farm is seasonal. In this population, given the seasonality and limited duration of access to home-produced maize, QPM may have limited impact on the nutritional status of infants and young children, even if their diets are deficient in protein and there is high adoption and utilization of the crop. Furthermore, the prevalence of inadequate intakes appears to increase as access to home production decreases, indicating that the potential impact of a biofortified crop may be more limited just as the need for it increases.

Successful targeting of QPM or other biofortified crops will require data on the diets of target populations, which often include infants and young children, and on access to home production of the target crop, recognizing that both diets and access are seasonal. The full diet must be assessed, not just consumption of the target crop, with consideration of other dietary sources of the target nutrient. For infants and young children, information on breastfeeding practices is also relevant and useful. Promotion and extension efforts may be able to address strategies, such as use of agricultural inputs to increase production or increased access to improved storage technologies, that will increase access and duration of access to biofortified crops in target populations. Complementary interventions integrated into biofortification programs may also be useful in modifying household behaviors to increase potential impact. These programs should also explore strategies for at-risk populations to access biofortified crops and foods through markets as well as home production. However, in the case of QPM and other crops with improvements in nutritional quality that are not visually detectable, it may be difficult to guarantee their nutritional quality when purchased in local rural markets.

## Figures and Tables

**Table 1:** Characteristics of households and children at the first assessment (post-harvest, *n* = 218).

Characteristic	Mean	Standard Deviation (SD)
Household size	5.9	2.2
Household head’s education (years)	6.1	3.6
Caregiver’s education (years)	3.3	3.0
House has improved roof	45%	-
House has improved walls	18%	-
Household has own toilet	66%	-
Land (ha)	0.4	0.2
Maize area (ha)	0.3	0.2
Household grew vegetables	38%	-
Household grew coffee	59%	-
Household grew khat	69%	-
Week’s food primarily from home production	67%	-
Child is female	49%	-
Child age (months)	24.7	7.7
Currently breastfeeding	78%	-

**Table 2:** Children’s consumption of key food groups and dietary diversity [27] in a 24-h period at each assessment.

	% Children Consuming	
Food Group	Assessment 1	Assessment 2
Maize	95	96
Other grains, roots, and tubers	65	63
Legumes and nuts	2	6
Dairy products	68	61
Animal flesh foods	0	0
Eggs	0	0
Vitamin A-rich fruits and vegetables	40	48
Other fruits and vegetables	21	25
Dietary diversity (mean, SD)	2.3 (0.7)	2.4 (0.7)

**Table 3:** Contribution of maize to children’s diets from complementary feeding. Breastmilk intake is not included.

	Assessment 1				Assessment 2			
	Mean	SD	Min	Max	Mean	SD	Min	Max
Maize consumed (g/day)	130	66	0	286	120	53	0	272
Energy intake (kcal/day)	830	387	133	2232	635	222	113	1671
Energy from maize (kcal/day)	469	238	0	1034	369	175	0	835
Energy from maize (% of total)	59%	-	0	99%	61%	-	0	100%
Total protein intake (g/day)	18.0	7.7	3.4	51.7	13.8	4.5	2.8	27.7
Total protein from maize (g/day)	9.0	4.6	0.0	19.9	7.4	3.4	0.0	16.8
Total protein from maize (% of total)	51%	-	0	100%	55%	-	0	100%

SD: standard deviation; Min: minimum; Max: maximum.

**Table 4:** Total and utilizable protein intakes and protein inadequacy, accounting for breastfeeding and simulated substitution of conventional maize with quality protein maize (QPM).

		Complementary Foods Only				Complementary Foods + Breastmilk			
		No QPM		With QPM		No QPM		With QPM	
		**Mean**	**SD**	**Mean**	**SD**	**Mean**	**SD**	**Mean**	**SD**
Assessment 1	Total protein intake (g/day)	18.0	7.7						
	Utilizable protein intake (g/day)	13.1	7.1	14.4	6.9	16.6	6.8	17.8	6.6
	% children below average requirement	28%	-	17%	-	6%	-	5%	-
Assessment 2	Total protein intake (g/day)	13.8	4.5						
	Utilizable protein intake (g/day)	9.9	4.0	11.1	3.8	13.1	4.2	14.3	4.1
	% children below average requirement	38%	-	25%	-	12%	-	6%	-

## Supplementary Materials

The following are available online at http://www.mdpi.com/2072-6643/11/1/192/s1, Table S1: Essential amino acid content of conventional maize and “high-lysine maize” (adapted from Bressani 1991 [4]).
