# Supplementary material for: Biofortified Maize Can Improve Quality Protein Intakes among Young Children in Southern Ethiopia
Source: Nutrients. 2019 Jan 18;11(1):192. doi: 10.3390/nu11010192 (PMC6356635; doi:10.3390/nu11010192)
Supplement: Supplementary file 1 [file nutrients-11-00192-s001.pdf]

Table S1. Essential amino acid content of conventional maize and "high-lysine maize" (adapted from Bressani 1991 [4]).

Amino Acid	mg/g Nitrogen	
	Conventional Maize	High-lysine Maize
Lysine	177	256
Tryptophan	35	78
Isoleucine	206	193
Leucine	827	507
Total sulfur amino acids	188	188
Total aromatic amino acids	505	502
Threonine	213	199
Valine	292	298
Leucine/isoleucine ratio	4.01	2.63
